# Extended reality for procedural planning and guidance in structural heart disease – a review of the state-of-the-art

**DOI:** 10.1007/s10554-023-02823-z

**Published:** 2023-04-27

**Authors:** Natasha Stephenson, Kuberan Pushparajah, Gavin Wheeler, Shujie Deng, Julia A Schnabel, John M Simpson

**Affiliations:** 1 School of Biomedical Engineering and Imaging Sciences, King’s College London, London, UK; 2 Department of Congenital Heart Disease, Evelina Children’s Hospital, London, UK; 3 Technical University of Munich, Munich, Germany; 4 Institute of Machine Learning in Biomedical Imaging, Helmholtz Center Munich, Munich, Germany; 5 St Thomas’ Hospital, 3rd Floor, Lambeth Wing, SE1 7EH London, UK

**Keywords:** Extended reality, Virtual reality, Surgical planning, Catheter planning, Procedure guidance, Structural heart disease

## Abstract

Extended reality (XR), which encompasses virtual, augmented and mixed reality, is an emerging medical imaging display platform which enables intuitive and immersive interaction in a three-dimensional space. This technology holds the potential to enhance understanding of complex spatial relationships when planning and guiding cardiac procedures in congenital and structural heart disease moving beyond conventional 2D and 3D image displays. A systematic review of the literature demonstrates a rapid increase in publications describing adoption of this technology. At least 33 XR systems have been described, with many demonstrating proof of concept, but with no specific mention of regulatory approval including some prospective studies. Validation remains limited, and true clinical benefit difficult to measure. This review describes and critically appraises the range of XR technologies and its applications for procedural planning and guidance in structural heart disease while discussing the challenges that need to be overcome in future studies to achieve safe and effective clinical adoption.

**Supplementary Information:**

The online version contains supplementary material available at 10.1007/s10554-023-02823-z.

## Introduction

Structural heart disease has been defined as non-coronary cardiac abnormalities encompassing both congenital and acquired defects [1]. Complex spatial relationships and significant individual anatomical variation make planning catheter and surgical interventions challenging, both for initial, and subsequent procedures. Detailed planning using imaging data from echocardiography, computed tomography (CT) and magnetic resonance imaging (MRI) is the standard of care and has traditionally been based on two-dimensional (2D) planar reconstructions which aid the user to mentally reconstruct the patient’s anatomy. As the complexity of surgical and transcatheter intervention has increased so has the need for more advanced image display methods which give the user a better, more intuitive understanding of the patient’s intra- and extracardiac spatial arrangement.

Initially, volume- or surface-rendering techniques were used to create an illusion of depth within a 2D image using colour and lighting effects. In the past 15 years three-dimensional (3D) printing technology has enabled the creation of a 3D physical replica of the patient’s heart from cross-sectional imaging data with the ability to simulate procedures. However, the significant time and expense of creating models, the difficulty in segmenting finer cardiac structures such as valves, and inability to recreate dynamic motion, are key limitations. Extended reality (XR) data has the potential to provide accurate and detailed 3D image visualisation of multimodality imaging without these constraints.

This review summarises the current range of reported clinical applications and validation of XR technology with respect to procedural planning in structural heart disease, highlights the current limitations in both the technology and the current literature, and discusses scope for the future.

### Extended reality - definitions and technology

**Fig. 1:**
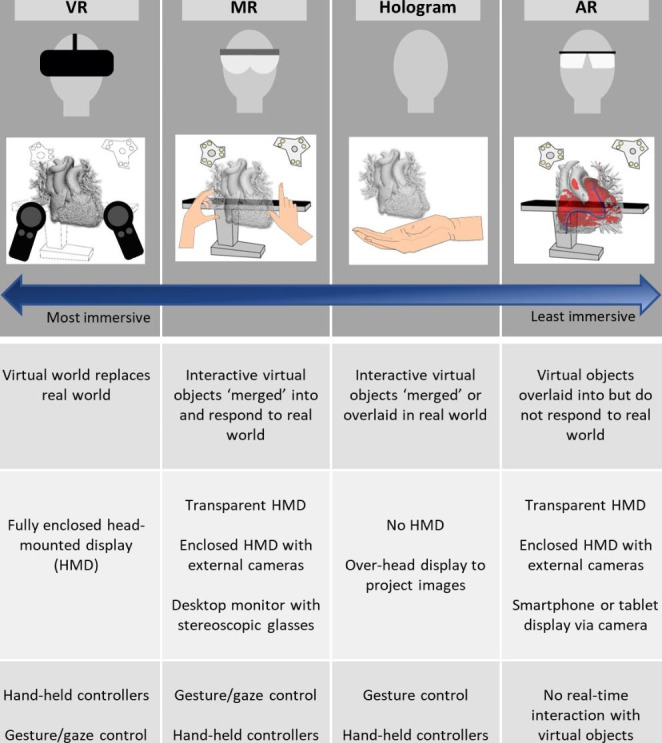
Pictorial representation of major XR modalities and key features

XR is an umbrella term that covers virtual reality (VR), mixed reality (MR) and augmented reality (AR) [2, 3]. The XR ‘spectrum’ is illustrated in Fig. 1. In this review, based on the publications analysed, VR will denote technologies where the real world is completely occluded and replaced with a virtual input, MR when virtual objects can interact with the user and the real-world environment, and AR when virtual information is visible but cannot respond to the user or environment.

Many XR visualisation systems are headsets that achieve 3D image display through stereoscopy, creating the impression of depth in the viewed image [4]. VR headsets are opaque, obscuring the outside world. Conversely, MR and AR headsets must show the user the outside world, and may achieve this in one of two ways. First, they may be transparent, allowing the user to see through to the real world. An image of the virtual objects is then superimposed upon this, for instance using optical waveguide technology [5]. Alternatively, they may use an opaque headset with cameras to provide a live video ‘feed’ of the user’s real surroundings, with virtual objects incorporated to create a merged environment. In VR and MR systems user interaction may be provided either by dedicated controllers, or by using hand and gaze tracking combined with gesture control.

Non-headset XR systems include stereoscopic flat monitor displays. These may require the user to wear special glasses, to provide an image with depth, and track their head position to provide an immersive window into a virtual world. Smartphones and tablets may also be used as AR systems, superimposing virtual objects upon the live video feed from their cameras. Digital holography, which differs from the stereoscopic displays described above, uses an ‘over the head’ display to project an interference-based volumetric hologram suspended in free space [6].

## Methods

A systematic search methodology was utilised in accordance with the Preferred Reporting Items for Systematic Reviews and Meta-Analyses guidelines [7]. A literature search was performed using Medline, Embase and the Cochrane library using a combination of search terms including all publications from inception until November 2021. The Medline and Embase search strategy are detailed in supplementary table S1 and was adapted for the requirements of the Cochrane library.

**Fig. 2:**
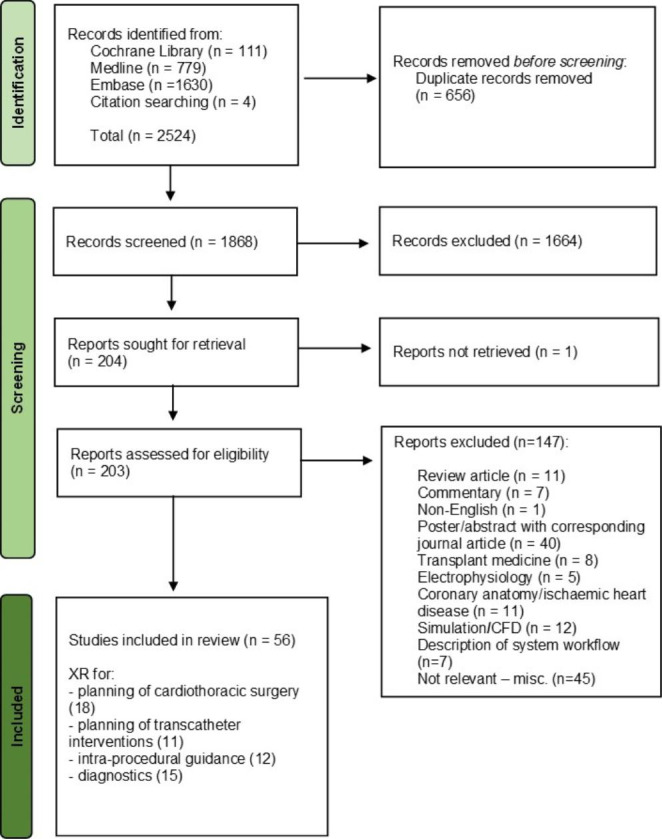
Flow chart demonstrating systematic literature search with inclusions and exclusions

After removal of duplicates, the title and abstract of each publication were reviewed to determine eligibility. Only publications written or translated into English which reported first-hand on results of a study investigating use of XR in diagnosis, procedural planning or guidance of structural heart diseases were included. Specific exclusions were (1) non-primary research or review articles; (2) subspeciality fields to which the search was not adequately directed, e.g. transplant, electrophysiology, ischaemic heart disease; (3) duplicate publications, in which case only the study reporting the largest dataset was included. The approach to identification of relevant papers is illustrated in Fig. 2. Included articles underwent full text review, and references cited in these studies were also examined for relevance using the same inclusion criteria. Full text publications of relevant studies were reviewed by one reviewer (N.S.). Two other authors (J.M.S. / K.P.) arbitrated on inclusion or exclusion.

## Results

56 studies were included in the final analysis. The included publications fell into four main categories – XR for planning cardiothoracic surgery, transcatheter interventions and device-sizing, intra-procedural guidance and diagnostics.

### Publication trends

#### Publications by year

**Fig. 3:**
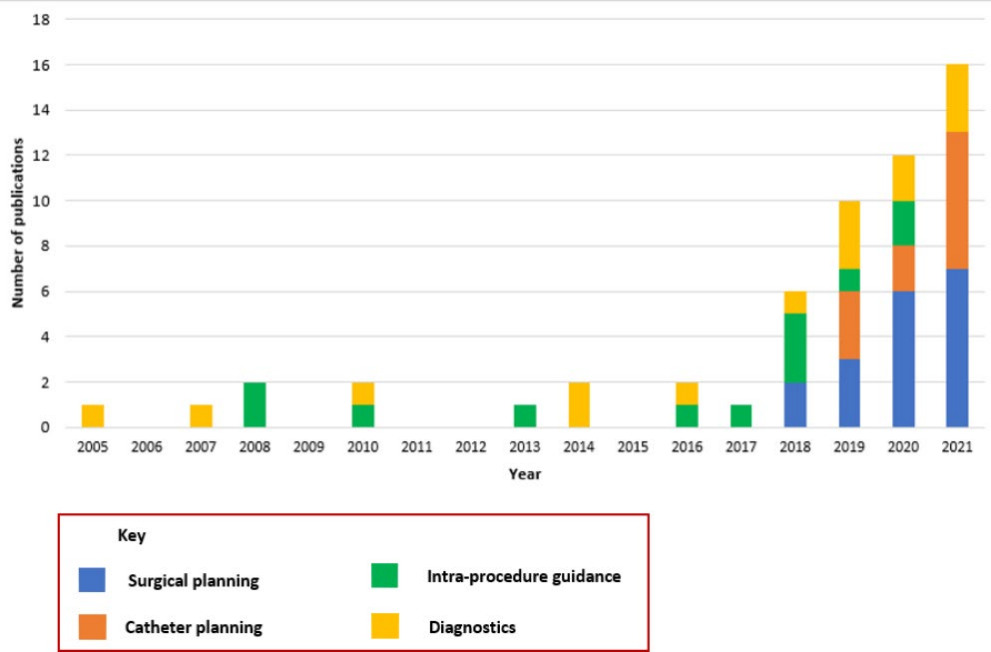
Bar chart demonstrating included publications by year and type of use

Between 2005 and 2017, 12 studies were published, with up to 2 publications each year during this time period. From 2018 until the end of 2021, 44 studies were published in this area, more than the total from the preceding 13 years. The number of annual publications has increased each year since 2017; from 6 to 2018 to 16 in 2021. This trend of rapidly increasing research interest is shown in Fig. 3.

#### Types of XR used

**Fig. 4:**
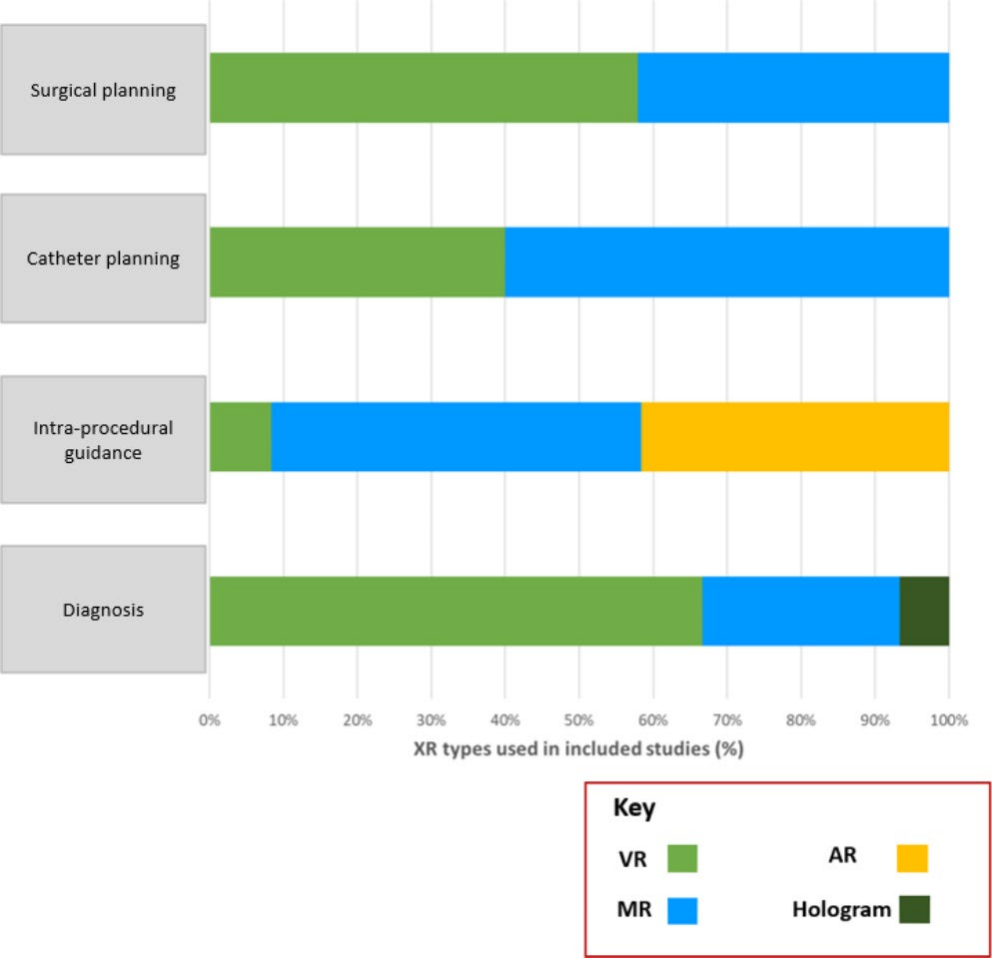
XR subtypes used in different study categories

VR is the most studied form of XR, with 28 included studies (50%) describing its use. MR also features prominently, with 22 publications (39%) using this modality, mostly published after 2018. There is clear predominance of MR and AR in real-time guidance of cardiac procedures, used in 93% of these studies (Fig. 4). Publications using XR for pre-procedural planning of catheter interventions also used MR systems most frequently (60%). In contrast, VR was the dominant modality used for surgical planning (58%) and diagnostics (67%).

At least 33 distinct XR systems were used, with some being used in more than one study. Seven studies used Echopixel’s True 3D Viewer, 5 Carnalife Holo, 3 Artiness Articor, two Vesalius 3D Stereo Viewer, two CardioVR and two used Barco i-Space. To the best of our knowledge, five XR systems had definitive approval as medical devices by major regulatory bodies at the time of writing (Echopixel’s True 3D Viewer, Carnalife Holo, Artiness Articor, Vesalius 3D Stereo Viewer and Realview Imaging).

#### Study methodology

**Fig. 5:**
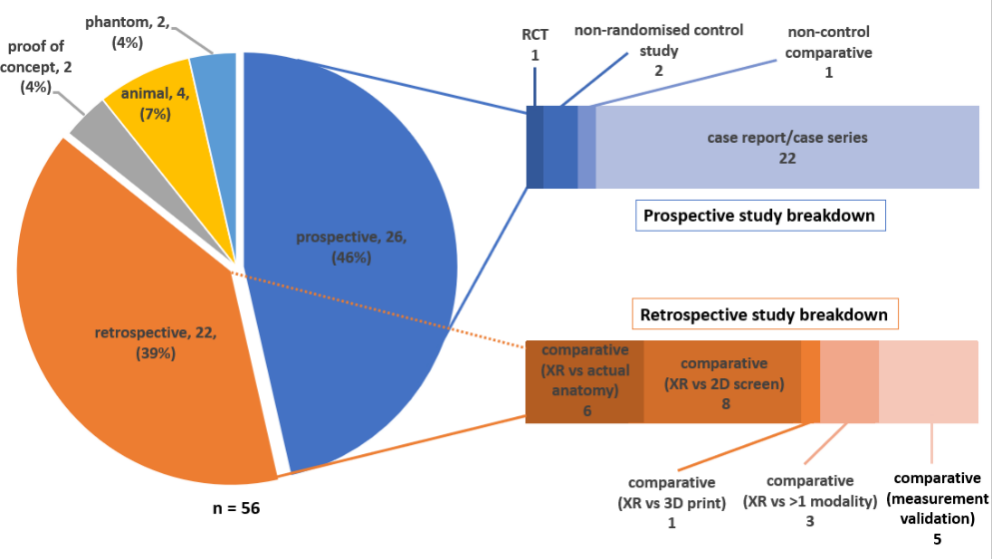
Graphical representation of study methodologies utilised in included studies

Twenty-six prospective studies were included in this literature review, accounting for 46% of all publications. Four of these prospective studies were comparative, comprised of one randomised-controlled study, 2 non-randomised control studies and one comparative study with no control group (Fig. 5). Twenty-two (85%) of the prospective studies were descriptive case reports or series. In the remaining 23 studies, a retrospective comparative methodology predominated, whereby XR visualisation was compared to standard visualisation of imaging on a 2D screen, to other 3D technologies or intra-procedural findings.

### Applications

#### XR for planning cardiothoracic surgery

Eighteen studies assessed XR for planning cardiothoracic surgery. Publications with 5 or more patients are summarised in Table 1.

Two experimental studies included a control group. Gehrsitz et al. evaluated the utility of photorealistic (cinematic) rendering displayed in MR in 26 patients undergoing a range of congenital cardiac surgeries, compared to retrospectively matched controls who had undergone surgery without MR review [8]. Intra-operative preparation time was significantly shorter in the study group (mean 58 min) compared to controls (mean 73 min). Surgeons preferred MR visualisation in most domains, but found it less useful for intracardiac anatomy, which the authors reported was due to the specific rendering technique. Ye et al. conducted a single-centre randomised study comparing XR to standard image visualisation in 34 patients with double-outlet right ventricle (DORV) [9]. The intervention group, where surgeons reviewed imaging in MR, had a significantly shorter planning time (mean 52 min) compared to the control group (66 min). Every patient in the MR group had their anatomy correctly identified pre-operatively with no change to the proposed surgical plan, whereas the surgical plan was altered in 3 control group cases due to unexpected intra-operative findings.

In other studies, benefit of XR was evaluated by surgeons reviewing imaging both virtually and on standard flat-screen software. Lu et al. asked surgeons to review data in MR as well as on 2D screens prior to performing a range of congenital cardiac surgeries [10], which led to alteration in surgical strategy in 2 cases (8%). Pushparajah et al. compared VR to flat-screen imaging review for planning atrioventricular (AV) valve surgery in 15 patients [11]. In 67% of cases, surgeons reported that VR gave them more confidence in the anatomy and they would have modified their surgical approach in nearly 60% of cases. Milano et al. found that surgeons could better delineate patient anatomy and provide an accurate surgical plan after reviewing DORV patients’ imaging in VR, compared to 3D-printed models or a 3D segmentation on a flat screen [12]. Vettukattil et al. used MR image visualisation to assess 7 patients’ suitability for biventricular repair after previously being considered either inoperable or suitable only for univentricular palliation [13]. All patients successfully underwent biventricular repair with no mortality in a mean follow-up period of 22 months. Pather et al. compared VR to 3D prints and flat screen review [14], with structured feedback suggesting that clinicians assessed 3D prints and VR as superior to 2D visualisation and VR had the most potential benefit for surgical planning.

The majority of prospective studies were case reports or series describing feasibility of XR for surgical planning and obtaining structured feedback from surgeons on perceived usefulness of XR. Larger case series included XR for planning surgery in 5 cases of pulmonary atresia and major aortopulmonary collaterals (MAPCAs) [15], complex aortic valve reconstruction in 26 patients unsuitable for valve replacement [16], 17 pulmonary artery reconstructions and unifocalisations [17], and 6 complex redo and minimally invasive surgeries in adults [18]. In seven case reports XR was used for planning repair of DORV [19], truncus arteriosus [20], a complex ventricular septal defect (VSD) [21], right ventricular to pulmonary artery conduit stenosis and left ventricular outflow tract (LVOT) obstruction [22], tetralogy of Fallot (TOF) [23], and resection of a large cardiac tumour [24]. In all studies, physicians and surgeons reported subjective benefit of XR visualisation for surgical planning. Although not yet trialled clinically, Vigil et al. described a workflow for VR intracardiac baffles which could be inserted within MRI-derived models, and which the authors postulated may be beneficial for patients with DORV undergoing complex biventricular repairs [25].

**Table 1:** Studies assessing XR for surgical planning (excluding case series/reports with fewer than 5 patients)

First author	Year	Cohort	Lesion(s)	Pt no.	Control	XR type	Modality	Study description	Results
Sadeghi et al.[17]	2020	Adult	TV repair, Ao root/arch reconstruction, VAD extraction	6	N	VR	CT	Prospective case series Qualitative surgeon evaluation of VR system in procedure planning.	Perceived user-friendliness 4/5; usefulness and efficiency 4.4/5; attitude towards (future) use 4/5
Lu et al.[10]	2020	Paed.	AVV repair, VSD closure, DORV repair, TAPVD repair/revision, MAPCA unifocalisation, LSVC baffling to RA	25	N	MR	echo, CT, MRI	Prospective comparative study Qualitative surgeon feedback on prospective use of MR system for surgical planning compared to 2D screen	MR images reviewed for longer (8 vs. 3 min, p < 0 0.001) MR review “worthwhile” in 96% cases Improved anatomical understanding in 84% Surgical plan altered in 2 cases
Cen et al.[15]	2021	Paed.	PA + MAPCAs	5	N	VR/ MR	CT	Prospective case series - surgeon review of 3D print and VR of segmented model STL pre-operatively, intraoperative display of the model in MR and questionnaire	No mortality. 3 complications - prolonged pleural drainage, ST changes and pneumonia. Surgeons reported subjective benefit of all 3D modelling modalities.
Tedoriya et al.[26]	2020	Adult	AoV repair	26	N	MR	CT	Prospective case series – review of CT imaging in VR prior to AoV repair	6/26 required additional procedure 1/26 required AVR 1/26 died 19/26 - good echocardiographic result
Vettukattil et al.[13]	2020	Paed.	TAPVD + AVSD (n = 4) ccTGA + PS PA/IVS + failing Fontan Univentricular + PAB	7	N	MR	CT	Prospective case series – MR review of imaging to determine feasibility for biventricular repair	Biventricular repair in 7/7, no mortality. 4/7 - uncomplicated recovery 2/7 – required ECMO 1/7 – AKI and Guillain-Barre. Clinical status at 11 months − 5/7 NYHA I, 1/7 NYHA II, 1/7 ongoing recovery from Guillain-Barre.
Ye et al.[9]	2021	Paed.	DORV	34	Y	MR	CT	Prospective randomised control study Patients allocated to pre-op imaging review on 2D screen (control) or standard + MR review imaging (intervention).	Surgical planning time reduced in MR group (52 ± 11 min vs. 66 ± 18 min; p < 0.05) Correct pre-op identification of anatomy in all MR cases, incorrect in 2 control group cases No change to pre-op plan in MR group, strategy modified in 3 control cases.
Gehrsitz et al.[8]	2021	Paed.	TOF, CoA, AP window, ALCAPA, TGA, PV disease, PA/VSD, truncus arteriosus, ccTGA, AVSD, ductus arteriosus aneurysm	26	Y	MR	CT, MRI	Prospective comparative study - surgeons completed structured questionnaires comparing 2D screen imaging review, MR, and 3D-printed model. Surgical preparation time compared with retrospectively matched controls.	MR rated better than 2D-monitor imaging + 3D prints in all categories. (mean 4.4/5 ± 1 vs. 3.7/5 ± 1.3, *p* < 0.05). 3D print + MR reduced intra-op preparation time (59 ± 23 min vs. 73 ± 43 min, P < 0.05).
Chan et al.[18]	2021	Paed.	Unifocalisation of MAPCAs, pulmonary artery reconstructions	17	N	MR	CT	Prospective case series – MR review of segmented CT models prior to surgery.	No system-related surgical complications Manual image processing time 2–4 h MR viewing time from 10–30 min
Pushparajah et al.[11]	2021	Paed.	AVV repair	15	N	VR	3DE	Retrospective – surgeon review of pre-op 3DE from previous AVV surgery. Review on 2D screen and in VR. Recommended surgical strategy recorded for 2D + VR review, compared to operation note	In 67% of cases, the surgeon reported that VR gave them more confidence in the anatomy and would have made modifications to surgical approach in over 57% of cases.
Milano et al.[12]	2019	Paed.	DORV	10	N	VR	CT, MRI	Retrospective – surgeons reviewed segmentations on 2D screen, 3D print, and in VR. Recommended suitability for biventricular repair and requirement for ASO	Surgical strategy correctly identified in 70% after 2D review, 85% after 3D print and 95% after VR visualisation. Correctly identified need for ASO in 45% with 2D review; 55% with 3D print and 60% after VR review

Paed. : paediatric, N: no, Y: yes

3DE: 3D echocardiography, AKI: acute kidney injury, ALCAPA: anomalous origin of the left coronary artery from the pulmonary artery, Ao: aorta, AP: aortopulmonary, AVSD: atrioventricular septal defect; AVR: aortic valve replacement, AVV: Atrioventricular valve, ccTGA: congenitally-corrected transposition of the great arteries, CoA: coarctation of the aorta, DORV: double outlet right ventricle, ECMO: extracorporeal membrane oxygenation, LSVC: left superior vena cava, MAPCA: major aortopulmonary collateral, PA: pulmonary atresia; PAB: pulmonary artery band, PV: pulmonary valve, RA: right atrium, TOF: tetralogy of Fallot, TV: tricuspid valve, VAD: ventricular assist device, VSD: ventricular septal defect

#### XR for planning transcatheter intervention and device implantation

Four studies assessed XR image visualisation for planning catheter interventions (Table 2). Four case reports and 3 proof-of-concept studies were also identified. A large experimental study compared CT-derived segmentations visualised in MR to traditional procedure planning using 2D transoesophageal echocardiographic (TOE) to plan transcatheter left atrial appendage occlusion (LAAO) [27]. There was a statistically significant reduction in total procedure time and device wastage, with a trend towards lower contrast use in the XR group. Two retrospective studies assessed XR for sizing of valves or devices. Sinha et al. found no significant difference between MR-derived annulus measurements and implanted valve size in 38 patients who had previously undergone TAVI, and concluded that MR may improve landmark identification and assist sizing of valves [28]. Another group used a VR system to predict mitral and aortic paravalvar leak occluder sizing [29], and although they found device size most closely approximated measurements made on 2D CT images, feedback was that VR was the most helpful tool for assessing the 3D nature of the leak. Tandon et al. used segmented CT datasets of 28 patients with superior sinus venosus atrial septal defect (SVASD) in VR to determine suitability for transcatheter closure [30]. They incorporated cylindrical stent models into the system which enabled assessment of potential obstruction of anomalous pulmonary veins.

The case reports described use of XR for successful planning of a challenging SVASD case [31], transcatheter Fontan completion [32], systemic venous baffle obstruction post-atrial switch [33], and hybrid approach in multiple muscular VSDs [34]. Three further studies described workflows and feasibility for device incorporation or sizing in LAAO [35], and septal occluders in VSDs prior to transcatheter intervention [36], but were not tested clinically in these publications.

**Table 2:** Studies using XR to plan catheter intervention and device implantation (excluding case series/reports with fewer than 5 patients)

First author	Year		Cohort	Lesion(s)	Pt no.	Control	XR type	Modality	Study description	Results
Dutcher et al.[27]	2020	Adult		LAAO	154	Y	MR	CT	Prospective control study Pre-op imaging review in MR (intervention) or 2D TOE (control) for patients undergoing LAAO.	Correct device selection first-time more frequent in MR group (86.7% vs. 75.6%; p = 0.041). Average procedure time reduced in MR group (33.6 min vs. 46.5 min; p < 0.001). Trend toward lower contrast dose in MR group (not statistically significant)
Sinha et al.[28]	2019	Adult		TAVI	38	N	MR	CT	Retrospective MR system used to calculate annulus diameter and compared to size of valve implanted.	No significant difference between calculated diameter and actual implant valve size (p = 0.41)
Sadeghi et al.[29]	2021	Adult		Paravalvar leak occlusion (AoV, MV)	6	N	VR	3DE, CT	Retrospective Comparison of VR review to 2D CT review (2DCT) and segmentation on 2D screen (3DCM) for sizing of paravalvar leak occluders	Similar measurements obtained from 3DCM models and VR. 2DCT measurements closest to actual device dimension
Tandon et al.[30]	2019	Adult		Superior SVASD	28	N	VR	CT, MRI	Retrospective case series VR for planning transcatheter stenting of SVASD.	6/28 unsuitable for transcatheter repair because of a large aPV 4/28 unsuitable for other reasons 7/28 equivocal as only small aPV would be blocked. Strong correlation between predicted stent size and SVC diameter but did not consistently predict stent size

3DE: 3D echocardiography, AoV: aortic valve, aPV: anomalous pulmonary vein, LAAO: left atrial appendage occlusion, MV: mitral valve, SVASD: sinus venosus ASD, TAVI: transcatheter aortic valve implantation, TOE: transoesophageal echocardiography

#### XR for intra-procedural guidance of cardiac interventions

Larger studies assessing XR for intra-procedural guidance are summarised in Table 3. Prior to use in humans, a number of systems which use XR guidance with catheter electromagnetic tracking systems have been tested in non-human models. James et al. demonstrated superiority of their VR system for guiding transseptal puncture in a cardiac phantom compared to fluoroscopy [37]. Another group published a series of feasibility studies testing AR intra-procedural guidance for minimally-invasive cardiac surgery in phantom and animal models. In a cardiac phantom, they demonstrated similar precision of AR and fluoroscopy for TAVI valve deployment by a single operator [38]. The system has also been tested in a live porcine model to demonstrate feasibility of ultrasound-guided minimally invasive mitral valve replacement[39], atrial septal defect (ASD) closure [40, 41], and neochordae prosthesis insertion for flail mitral leaflets [42], with small studies showing that AR guidance could reduce procedure time and the risk of injury to cardiac structures by improving navigational accuracy.

At the time of writing, there are no controlled experimental studies using XR for intra-procedural guidance in cardiac structural interventions. A proof-of-concept study demonstrated that an MR guidance system enabled placement of cerebral embolic protection (CEP) devices in 6 patients undergoing TAVI [43], eliminating the need for aortic arch angiography prior to filter placement. Another group published case reports describing use of their MR system for intra-procedural guidance in a range of cardiac catheter procedures including patent ductus arteriosus (PDA) device occlusion[44], TAVI [45], pulmonary artery interventions [46], balloon mitral commissurotomy [47], and LAAO [48].

**Table 3:** Studies using XR for intra-operative guidance (excluding case reports or series with fewer than 5 patients)

First author	Year	Cohort	Lesion(s) / procedure	Pt no.	Control	XR type	Modality	Study description	Results
James et al.[37]	2020	Phantom	Simulated TSP	NA	Y	VR	MRI	Feasibility study Simulated TSP by 8 participants on a cardiac phantom using fluoroscopy (control) or VR guidance (intervention)	VR guidance more accurate (3.5 ± 3 mm vs. 10 ± 10 mm; p = 0.01; from fossa centre) Longer distance travelled by needle with VR (mean 22.5 cm vs. 20.5 cm; p = 0.04) No difference in procedure time
Vasilyev et al.[41]	2008	Animal	ASD	6	Y	AR	echo	Feasibility study Real-time AR stereoscopic guidance compared to standard echo guidance in an animal model of minimally-invasive ASD closure.	Reduced mean deployment time in AR group (9.7 ± 0.9s vs. 17.2 ± 0.9s; p < 0.001). Improved navigational accuracy of catheter tip in AR group: 3.8 ± 0.7 mm vs. 6.1 ± 0.3 mm, p < 0.01. Accuracy of anchor placement not significantly different between groups
Guiraudon et al.[39]	2010	Phantom Animal	MV replacement	12	Y	AR	echo	Feasibility study 3D AR guidance system vs. standard 2DE guidance for minimally-invasive MV replacement system in phantom and animal model	AR guidance improved positioning of MV system (error 0.99 mm ± 0.4 mm vs. 4.96 ± 2.3 mm, p < 0.05) in phantom
Sadri et al.[43]	2018	Adult	CEP filter placement in context of TAVI	6	N	MR	CT	Case series Use of intra-procedure CEP filter placement using MR review of CT models.	CEP filters placed successfully in all patients. MR guidance eliminated need for arch angiograms and additional contrast prior to CEP filter placement.

NA – not applicable

2DE: 2-dimensional echocardiography, ASD: atrial septal defect, CEP: cerebral embolic protection, MV: mitral valve, TAVI: transcatheter aortic valve implantation, TSP: trans-septal puncture.

#### XR as a diagnostic tool

Fifteen studies assessed XR for its diagnostic capabilities for structural heart lesions, nine of which included more than 5 patients (Table 4). The most frequent application was for assessment of AV valve morphology and function. A Dutch group performed 2 small studies assessing an early VR system to assess AV valve function [49, 50]. They showed that it was possible to differentiate normal from abnormal valves, and appreciate additional information in VR which had not been delineated on standard 2D assessment. Beitnes et al. assessed diagnostic yield of VR in mitral valve prolapse [51], and demonstrated > 90% sensitivity and specificity compared to intra-operative findings. In addition to assessing anatomy, XR was also assessed for measurement accuracy and precision. Measurements in echocardiographic data from normal and diseased mitral valves were assessed in an industry-standard semi-automated mitral modelling system and in MR [52]. Overall, agreement between systems was better for normal than pathological valves, which the authors attributed to the loss of normal valve architecture making measurement more challenging. Another group assessed measurements on 3DE images of a phantom and AV valves in VR against two industry standard platform [53]. VR was determined to be more accurate but less precise than standard software, the latter attributed to lack of familiarity with their system. Two other publications demonstrated that VR display reduced AV valve measurement variability and improved repeatability compared with standard software [54, 55].

XR visualisation was also assessed for the identification of septal defects [56, 57], DORV [56, 58], MAPCAs[59], precise aortic root anatomy [60], and complex congenital lesions [61]. In all studies they were either equivalent or superior to standard methods of image display for detection of landmarks or lesions. In one study, the interpretation time was also significantly reduced [59]. Kim et al. compared VR and non-immersive displays for CHD diagnosis in cardiology trainees, determining that accuracy was higher using immersive VR and that this bias increased with more complex lesions [62]. Two publications also provided a comparison of XR display to 3D-printed models: Raimondi et al. found that a senior cardiac surgeon’s diagnostic interpretation of complex CHD cases was equal or better using VR compared to a 3D printed model [61]. Based on structured feedback from 35 participants, Lau et al. concluded that 3D printed models and VR projection of segmentations were similarly effective in conveying anatomical information [63].

**Table 4:** Studies using XR for diagnostics including measurement (excluding case reports or series with fewer than 5 patients)

First author	Year	Cohort	Lesion(s) / Procedure	Pt no.	Control	XR type	Modality	Study description	Results
Van den Bosch et al.[49]	2005	NS	MV prolapse, stenosis, HCM with SAM of MV, LAVV	6	N	VR	3DE	Retrospective/proof of concept 10 observers reviewed 3DE images in VR and identify normal and pathological MVs	All correctly identified normal and pathological mitral valves.
Bol Raap et al.[50]	2007	Paed.	TV post-VSD closure	5	N	VR	3DE	Retrospective Observers reviewed 12 3DE datasets in VR for assessment of post-op TV function compared to 2DE	In 3 patients - VR analysis identified restriction of septal leaflet not appreciated on 2DE
Xue et al.[56]	2010	Paed.	ASD, VSD, TOF, DORV	40	Y	VR	3DE	Retrospective 3 observers reviewed datasets as 2DE, and as 3DE in VR. Asked to determine if abnormal intracardiac anatomy present. Compared to intra-op findings.	Diagnostic accuracy of VR > 92% Significantly higher accuracy in VR for TOF and DORV. ROC curve for VR closer to the optimal performance point than 2DE (AUC 0.96 in VR vs. 0.92 2DE)
Chan et al.[59]	2013	Paed.	PA + MAPCAs	9	N	MR	CT	Retrospective Participant identified MAPCA anatomy in standard 2D platform and in MR. Comparison made to gold standard (angiography).	Sensitivity, specificity, accuracy of standard CT review: 81%, 93%, 91% respectively; MR review 90%, 91% and 91%. Mean interpretation time shorter with MR: 13 ± 4 min vs. 22 ± 7 min (p = 0.0004)
Beitnes et al.[51]	2015	Adult	Degenerative MV disease	40	N	VR	3DE	Retrospective 2 observers assessed mitral valve segments in VR and compared to findings at intra-op inspection (35) or 3D TOE (5) as gold-standard.	Diagnosis sensitivity/specificity was 87/99% and accuracy/precision was 96/95% in VR Inter-observer agreement very good (Cohen’s Kappa 0.95)
Bruckheimer et al.[57]	2016	Adult /Paed.	ASD, percutaneous PVI, Glenn shunt	8	N	Holo-gram	3DE, RA	Proof of concept Assessment of assessing anatomy using 3D hologram in catheter lab. 4 observers asked to identify anatomical landmarks.	All anatomical landmarks identified by all participants. No adverse events reported.
Ballocca et al.[52]	2019	Adult	Degenerative MV disease	40	Y	MR	3DE	Comparison of measurements on normal and pathological mitral valves using standard 3DE platform and MR platform	Poor agreement between systems for abnormal MVs in annular area (ICC 0.58) & circumference (0.5). Good agreement in normal valves for annular area (0.95), circumference (0.91) and diameter (0.88–0.97). Poor agreement for scallop length in both normal and abnormal valves. Inter- & intra-observer agreement good (> 0.8) for all measurements except scallop length.
Wheeler et al.[53]	2019	Paed./ Phantom	Phantom, AV valves	5	Y	VR	3DE	Comparison of measurements in VR in phantom and patient data to 2 standard 3DE platforms	VR system more accurate in phantom and clinical measurements (lower mean difference) Precision higher in standard software (lower standard deviation)
Narang et al.[54]	2020	NS	Degenerative MV disease and MVR	30	N	VR	3DE, CT	Comparison of measurements in VR and conventional software Diagnostic quality of the VR models assessed	Measurement variability reduced in VR (20.1% standard vs. 12.2% in VR for 3DE; 15.3–10.1% for CT) Reduced measurement time (mean 61s in standard, 42s in VR for 3DE; 37s standard, 23 s in VR) for CT

NS: not stated, Paed.: paediatric.

2DE: 2 dimensional echocardiography, 3DE: three-dimensional echocardiography, ASD: atrial septal defect, AUC: area under the curve, AVSD: atrioventricular septal defect, CHD: congenital heart disease, DORV: double outlet right ventricle, LAVV: left atrioventricular valve, MAPCA: major aortopulmonary collateral artery, MV: mitral valve, MVR: mitral valve replacement; RA: rotational angiography, ROC: receiver operator characteristics, PA: pulmonary atresia, PVI: pulmonary valve implantation, TOF: tetralogy of Fallot, TV: tricuspid valve, VSD: ventricular septal defect.

## Discussion

Interest in XR for procedural planning in structural heart disease is rising, with a rapidly expanding literature base. The surge in research interest since 2018 mirrors XR technological improvements and increased availability over this time period. The launch of the recent generation of VR headsets and powerful graphics cards in 2016 significantly improved user experience via high quality images, realistic user interaction and reduced motion sickness. This set the scene for greater interest in the XR industry, with market projections estimating an increase in global market value from USD 11 billion in 2021 to 227 billion by 2029 [64]. Large industry players are investing heavily in XR, which will likely lead to greater accessibility, affordability and user-friendliness of equipment, and in turn increase its uptake in healthcare.

Review of the literature has indicated different use profiles for VR, MR and AR. VR continues to be the most-used type of XR at present in 50% of studies, followed by MR in 39%, AR in 9% and digital holography in 2%. VR was the dominant modality in surgical planning and diagnostics, probably because it completely immerses the user in the virtual environment, which may be advantageous for detailed image interrogation. Nonetheless, the enclosed headset isolates the user from their surroundings, and we found that studies using XR for real-time guidance strongly favour AR and MR, which enable the user to maintain sight and awareness of their real-world surroundings. Digital holography may also be applicable for live procedure guidance, although its dependence on bulky boom-mounted equipment makes it less practical.

Whilst holding much promise, AR and MR devices continue to have significant limitations such as a narrow field of view, limited battery life and suboptimal resolution (screen door effect), and technical improvements will likely be required before they are widely implemented in the clinical environment. In addition, an issue facing all current XR modalities is the lack of ability to share the full XR experience with a wider group of viewers without the requirement for multiple headsets. Whilst 3D screens and projectors hold some promise, the technology requires further development to enable the XR experience to be shared in complex case reviews or surgical conferences, which are mainstays in the multidisciplinary care of patients with structural heart disease. A further obstacle which may limit widespread adoption of XR in procedural planning is its clinical evaluation in high-quality studies. Structural and congenital heart disease comprises a small and hugely variable study population and obtaining an adequate sample size to achieve statistical power is difficult. And these difficulties are reflected in of the included publications. There was only one well-designed outcome study which was able to assess for incremental benefit of XR technology over standard imaging, albeit in a single centre and with small patient numbers [9]. This study demonstrated a reduction in surgical planning time of 14 min and improved pre-operative identification of anatomy using XR compared with flat screen display. Other prospective studies were liable to confounding due to use of different imaging modalities in the XR and standard groups and potential bias from use of retrospectively matched controls [8, 27]. Many studies relied either partly or entirely on subjective feedback from users. The vast majority of studies using XR for structural heart procedural planning were case reports or series, and to date there have been only case reports using XR intra-procedural guidance in humans. None of the included studies were designed or powered to assess for superiority of XR visualisation on patient outcome such as complication rate, length of stay or re-intervention rate. Nonetheless, there are some promising results with respect to improved operator confidence, timesaving, reduced device wastage and contrast use, all of which could improve patient outcomes and reduce healthcare costs.

Based on our results, XR has wide applicability within structural and congenital heart diseases, especially when planning procedures where understanding of complex anatomy is required such as for double outlet right ventricle repair, unifocalisation of MAPCAs or surgical or transcatheter repair of sinus venosus atrial septal defects. In addition to complex congenital lesions, this technology lends itself particularly well to visualising 3D interactions and may have value for planning adult structural interventions such as transcatheter valve implantations, appendage occlusion or intervention on the mitral and tricuspid valves. This has the potential to reduce complications such as compression of adjacent structures such as coronaries, pulmonary veins or the neo-left ventricular outflow tract as well as worsening of atrioventricular valve regurgitation. In future, multi-centre collaborations will be essential to enable clinically meaningful outcome measures to be adequately assessed and further expansion into the larger adult structural field could also enable this.

The majority of studies in this review describe the use of XR by surgeons and interventionists as an alternative or adjunct to ‘flat screen’ review with a radiologist or an imaging cardiologist. Structured feedback from many of the included studies suggests that XR lends itself well to use by this cohort given that the interface with imaging is generally more intuitive and enables tailoring of the individual’s experience [14, 54, 58, 63]. Incorporation of devices or patches into the XR environment may also enable clinicians to “road test” a procedure, reduce device wastage and potentially reduce procedure time or contrast use [29, 30, 36]. In this review a smaller selection of studies included radiologists or imaging cardiologists, however, feedback and results were largely positive. Additionally, implementation of novel volume-rendering and automated segmentation techniques into XR solutions could circumvent time-consuming manual segmentation and reduce workload [65].

Whilst XR is unlikely to replace standard 2D screen review in the clinical workflow, it has the potential to become a valuable tool in the arsenal of imaging specialists who have a crucial role in decision-making for patients. XR is most likely to become an adjunct to assist planning the more complex, high-risk cases where potential benefit is highest. It is likely that adoption beyond congenital heart disease would be required to stimulate technological development and investment by industry. Such potential benefits are not limited to cardiac disease, and many groups in other surgical disciplines such as maxillofacial, orthopaedics, neurosurgery and urology also investigating XR as a planning and guidance tool [66–69].

A lack of consistent regulatory standards and guidance is a challenge facing roll-out of this technology in the clinical arena [70]. Although international bodies have published standards for image quality assessment for near-eye displays, there are currently no validation standards which cover the heterogeneous and rapidly expanding range of hardware and software used in XR, nor of the interaction between them [71, 72]. A few groups have published their individual workflows and rendering pipelines, opening avenues for peer review and external validation, though this is not common practice [36, 73]. For an XR planning system to become approved as a medical device requires regulatory approval by country-specific authorising bodies, and requires individual review of the device’s safety profile including risk assessment, verification, validation and clinical evaluation data. From our review, only 5 of the included XR systems had definite regulatory approval and indicates that this process is challenging and may not be being consistently applied even in prospective studies impacting patient care. Standardisation of guidance and frameworks by the various authorising bodies could enable a more consistent approach to the evaluation of XR planning systems, and would promote multi-centre studies to evaluate their clinical efficacy and safety.

## Conclusion

The potential of XR to assist with planning and guiding cardiac procedures is beginning to be realised, with many researchers focusing on this area as an alternative or adjunct to other 3D technologies. Recent advances in computer power and headset technology mean it is now often compact enough for use in the clinical setting while still providing excellent image quality. This review has shown promising work suggesting that XR can enable intuitive understanding of 3D anatomy, and is a feasible option to assist with planning and guiding complex cardiac surgeries and interventions. Further work is required to demonstrate patient benefit or superiority to existing image visualisation systems. Given the increasing range of hardware and software options, as well as ongoing technical innovation, frameworks and standards from regulatory bodies could assist developers to align validation and clinical evaluation efforts and lead to more widespread adoption.

## Electronic supplementary material

Below is the link to the electronic supplementary material.

Supplementary Material 1

## Abbreviations

2D: Two-dimensional
3D: Three-dimensional
3DE: Three-dimensional echocardiography
ALCAPA: Nomalous origin of the left coronary artery from the pulmonary artery
AKI: Acute kidney injury
AR: Augmented reality
ASD: Atrial septal defect
AVSD: Atrioventricular septal defect
AVR: Aortic valve replacement
AVV: Atrioventricular valve
ccTGA: Congenitally-corrected transposition of the great arteries
CEP: Cerebral embolic protection
CHD: Congenital heart disease
CoA: Coarctation of the aorta
CT: Computed tomography
DAA: Double aortic arch
DORV: Double-outlet right ventricle
ECMO: Extracorporeal membrane oxygenation
LAA: Left atrial appendage
LAA: Left atrial appendage
LAAO: Left atrial appendage occlusion
LV: Left ventricle
LVOT: Left ventricular outflow tract
MAPCA: Major aortopulmonary collateral artery
MRI: Magnetic resonance imaging
PA: Pulmonary atresia
PAB: Pulmonary artery band
PDA: Patent ductus arteriosus
PDA: Patent ductus arteriosus
SVASD: Sinus venosus atrial septal defect
SVC: Superior vena cava
TAPVD: Total anomalous pulmonary venous drainage
TAVI: Transcatheter aortic valve implantation
TGA: Transposition of the great arteries
TOF: Tetralogy of Fallot
TOE: Transoesophageal echocardiography
VAD: Ventricular assist device
VR: Virtual reality
VSD: Ventricular septal defect
XR: Extended reality
