# Supplementary material for: Extended reality for procedural planning and guidance in structural heart disease – a review of the state-of-the-art
Source: Int J Cardiovasc Imaging. 2023 Apr 27;39(7):1405–19. doi: 10.1007/s10554-023-02823-z (PMC10250452; doi:10.1007/s10554-023-02823-z)
Supplement: Supplementary file 1 — Supplementary Material 1 [file 10554_2023_2823_MOESM1_ESM.docx]

**Extended reality for procedural planning and guidance in structural heart disease – a review of the state-of-the-art**

**Supplementary information (SI)**

**Submitted to:** The International Journal of Cardiovascular Imaging

**Authors:**

Dr Natasha Stephenson, BMedSci, MBChB (Hons), MRCPCH^1,2^

Dr Kuberan Pushparajah , BMedSci, BMBS (Hons), MDRes, MRCPCH^1,2^

Dr Gavin Wheeler, BE (Hons), PhD^1^

Dr Shujie Deng, BEng, MSc, PhD^1^

Professor Julia A Schnabel, PhD^1,3,4^

Professor John M Simpson, BSc, MBChB, MD, FRCP^2^

1. School of Biomedical Engineering and Imaging Sciences, King’s College London, United Kingdom
2. Department of Congenital Heart Disease, Evelina Children’s Hospital, London, United Kingdom
3. Technical University of Munich, Germany
4. Institute of Machine Learning in Biomedical Imaging, Helmholtz Center Munich, Germany

**Correspondence Address:**

Dr Natasha Stephenson

3rd Floor, Lambeth Wing, St Thomas’ Hospital, London, SE1 7EH

Email: Natasha.stephenson@kcl.ac.uk; Telephone: +447812375834

ORCID ID: 0000000252785271

**Table S1.** Search strategy

| Virtual reality/ (MeSH) OR virtual realit*.mp |
| --- |
| Extended realit*.mp |
| Augmented reality/ (MeSH) OR augmented realit*.mp |
| Mixed realit*.mp |
| VR.mp |
| Virtual 3D.mp OR virtual three dimensional.mp |
| Stereoscop*.mp |
| Holography/ (MeSH) OR hologra*.mp |
| 3D technology.mp |
| Immersive 3D visuali#ation.mp |
| 1 OR 2 OR 3 OR 4 OR 5 OR 6 OR 7 OR 8 OR 9 OR 10 |
| Heart Defects, Congenital/ (MeSH) OR congenital heart disease*.mp OR congenital heart defect*.mp OR congenital heart malformation*.mp Or p?ediatric heart.mp |
| Structural heart disease*.mp OR structural heart defect*.mp OR Valv*.mp |
| Double Outlet Right Ventricle/ (MeSH) OR double outlet right ventricle.mp |
| Pulmonary atresia/ (MeSH) OR Pulmonary atresia.mp |
| Heart septal defects, ventricular/ (MeSH) OR septal defect*.mp OR Heart septal defects, atrial/ (MeSH) OR Atrioventricular.mp OR sinus venosus.mp |
| Cardiac surgical procedures/ (MeSH) OR cardiothoracic surger*.mp OR cardiac surgery.mp |
| Cardiac catheterization/ (MeSH) OR cardiac catheter*.mp OR transcatheter.mp OR percutaneous intervention*.mp |
| Pre?operative plan*.mp OR procedur* plan*.mp |
| 12 OR 13 OR 14 OR 15 OR 16 OR 17 OR 18 OR 19 |
| 11 OR 20 |

Key to syntax used:
 * after a word is used as a truncation (“wildcard”) to retrieve plurals or different endings, e.g. stereoscop* would retrieve ‘stereoscopy’ and ‘stereoscopic’

? is an optional wildcard character which can be used within or at the end of a search term to substitute for one or no characters, e.g. pre?operative would retrieve ‘pre-operative’ or ‘preoperative’.

# is a mandated wildcard character which can be used within or at the end of a search term to substitute for different characters, e.g. visuali#ation retrieves ‘visualisation’ and ‘visualization’
 MeSH Medical Subject Heading
 / at the end of a phrase, searches the phrase as a subject heading
 .mp mapping alias (searches title, abstract, heading words, table of contents and key phrase identifiers)
